# Abiotic Stress Tolerance in Foxtail Millet (*Setaria italica* L.): From Molecular Mechanisms to Climate-Resilient Breeding

**DOI:** 10.3390/plants15101474

**Published:** 2026-05-12

**Authors:** Hong-Jin Wang, Xiangwei Hu, Yun Zhao, Baoyi Yang, Hui Wang, Jianan Huang, Qadir Bakhsh, Zaituniguli· Kuerban, Guojun Feng

**Affiliations:** 1 Crop Research Institute of Xinjiang Uygur Autonomous Region Academy of Agricultural Sciences, Urumqi 830002, China; wanghongjin@nwafu.edu.cn (H.-J.W.); huxw@xaas.ac.cn (X.H.); zhaoyun@xaas.ac.cn (Y.Z.); byyang@xaas.ac.cn (B.Y.); wanghui@xaas.ac.cn (H.W.); wangh@xaas.ac.cn (J.H.); 2 Institute of Plant Sciences, University of Sindh, Jamshoro 76080, Pakistan; qadirbakhsh054@gmail.com

**Keywords:** foxtail millet (*Setaria italica* L.), abiotic stress tolerance, signaling networks, reproductive development, breeding strategies

## Abstract

Abiotic stresses caused by climate change pose a significant challenge to global food security, making it necessary to develop stress-resistant crops. Foxtail millet (*Setaria italica* (L.) P. Beauv.) is a drought-tolerant C_4_ cereal and serves as a model crop for elucidating stress adaptation mechanisms and promoting climate-resilient agricultural solutions. This paper reviews the tolerance mechanisms of foxtail millet to abiotic stresses. Physiologically, the species exhibits excellent water-use efficiency, requiring 75% less irrigation than traditional cereals, achieved through enhanced osmotic adjustment via soluble substance accumulation and the maintenance of ion homeostasis. Morphological adaptations include reduced leaf area, adjusted stomatal density, well-developed root systems, and specialized anatomical features that optimize water conservation. At the molecular level, stress tolerance involves complex transcriptional networks mediated by multiple transcription factor family members, including those (*NF-Y*, *DREB*, *NAC*, *WRKY*, *MYB*) that coordinate stress-responsive gene expression, antioxidant defense systems, and osmotic adjustment pathways. Furthermore, this review summarizes multi-omics characteristics, including genomics (such as QTL mapping and GWAS), proteomics, transcriptomics, metabolomics, and regulatory networks, for foxtail millet under abiotic stress tolerance. Additionally, reproductive resilience is maintained through efficient mobilization of stem reserves to panicles, phenological plasticity in flowering timing, and preserved gametic viability under thermal stress. Combining advanced molecular breeding with the inherent tolerance of foxtail millet positions this crop as both a solution to climate change and a genetic resource for enhancing the stress resistance of other cereals. These findings establish foxtail millet as a valuable model for developing sustainable agricultural technologies essential for food security under projected climate scenarios.

## 1. Introduction

Worldwide agricultural productivity is increasingly threatened by the rapid development of climate change, and model studies predict that crop yields will decline significantly, thereby endangering global food security [1,2,3]. Abiotic environmental stresses, including salinity, drought, high and low temperatures, waterlogging, and nutrient deficiencies are major limiting factors for crop production. These stresses are particularly severe in arid and semi-arid agricultural ecosystems, where water resources are limited and soil degradation is widespread, hindering agricultural expansion [4,5,6,7]. Current estimates indicate that these abiotic stress factors reduce the annual yield of major food crops by 15–20%. Under multiple stress conditions, losses may exceed 50%, highlighting the urgency of developing climate-adaptive agricultural technologies [8,9].

Contemporary breeding programs face increasingly severe challenges because high-quality varieties optimized for favorable growth conditions perform poorly under the increasingly common multiple stress environments in modern agriculture [10,11,12,13]. This situation urgently calls for the identification and exploitation of stress-resilient crops with well-characterized molecular architectures suited for advanced genomic improvement [14]. Among underutilized yet climate-adaptive crops, foxtail millet, a member of the Paniceae tribe (subfamily Panicoideae, family Poaceae), has emerged as a key model for studying adaptation to abiotic stress. This cereal is valued for its stress resistance and genome characteristics that are suitable for modern breeding methods [15,16]. There are several compelling reasons for selecting millet as the focus of this review: (i) it is a C_4_ cereal, inherently superior in drought, salinity, and heat stress tolerance compared to major staple crops; (ii) it can grow on marginal lands with minimal inputs, making it directly relevant to food security in climate-vulnerable regions; (iii) it has a compact and fully sequenced diploid genome (490 Mb, 2n = 18), facilitating rapid functional genomics and gene editing; (iv) it is phylogenetically close to sorghum, maize, and sugarcane, allowing stress resistance mechanisms to be directly applicable to priority C_4_ crops [15,16,17,18]. Originating in China and domesticated about 8000 years ago, foxtail millet is widely cultivated across the Eurasian continent. As a diploid C_4_ grain, it has a compact genome of approximately 490 megabases (Mb) with a chromosome number of 2n = 18, and it contains about 38,801 protein-coding genes. It has a short growth cycle and exhibits strong innate tolerance to various abiotic stresses [15,16,17,18].

The availability of high-quality, well-annotated reference genome sequences [16,19,20] plays a crucial role. This foundational resource, combined with comprehensive analyses of gene families, collections of over 1500 germplasm materials covering global genetic diversity [21], and the development of stable transformation and gene editing methods [22,23], makes foxtail millet a powerful model system. These resources together facilitate comprehensive functional genomics studies, the results of which can be directly applied to the improvement of major food crops.

Foxtail millet exhibits excellent water use efficiency, requiring only 25–30% of the irrigation amount needed for traditional cereals such as rice (*Oryza sativa*), wheat (*Triticum aestivum* L.), and maize (*Zea mays*) [24,25,26]. This characteristic makes black millet very suitable for drought-prone areas. One of the key factors of its adaptability is its rapid 70–80-day life cycle, which allows for multiple cropping seasons. Therefore, this short growing period provides resilience for crops against increasingly threatening unpredictable weather, which has a greater impact on crops with longer growing periods [27].

The availability of multi-omics resources for specific species has greatly accelerated the elucidation of millets stress resistance. Whole-genome sequencing, QTL mapping, and genome-wide association studies (GWAS) have identified genomic loci and candidate genes in this species related to drought, salinity and heat resistance [26,28,29,30,31,32]. Under specific stress conditions, complementary analyses of the transcriptome, proteome, and metabolome have revealed the regulatory networks, post-translational modifications and metabolite changes driving adaptation [33,34]. Crucially, combining these millet-specific multi-omics data with morpho-physiological measurements allows molecular features to be directly linked to observable stress-resistance traits, providing actionable targets for breeding improvement [26,28,32,35].

**Figure 1:**
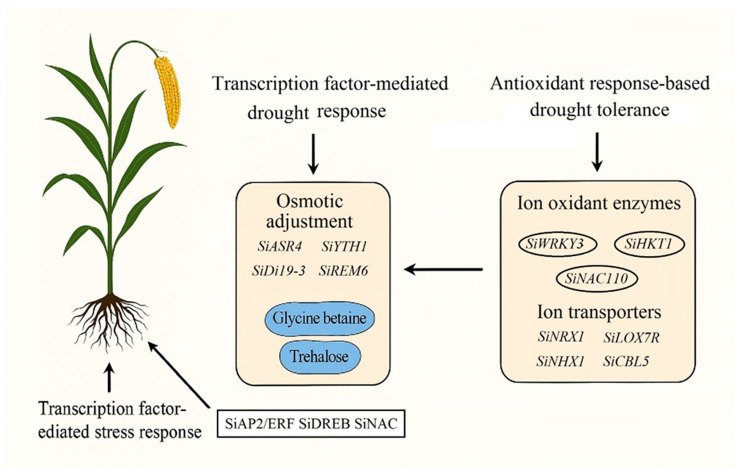
Molecular and physiological mechanisms of drought tolerance in foxtail millet. Drought stress activates two major response pathways: transcription factor-mediated osmotic adjustment (**left**) and antioxidant-based tolerance (**right**). The osmotic adjustment pathway involves activation of genes (*SiASR4*, *SiYTH1*, *SiDi19-3*, *SiREM6*) that produce osmoprotectants (blue ovals: glycine betaine and trehalose). The antioxidant pathway upregulates ion oxidant enzymes (*SiWRKY3*, *SiHKT1*, *SiNAC110*) and ion transporters (*SiNRX1*, *SiLOX7R*, *SiNHX1*, *SiCBL5*) to maintain redox balance and ionic homeostasis. Black arrows show regulatory relationships. Major transcription factor families (*SiAP2/ERF*, *SiDREB*, *SiNAC*) orchestrate these responses from the roots to coordinate whole-plant drought stress adaptation.

**Figure 2:**
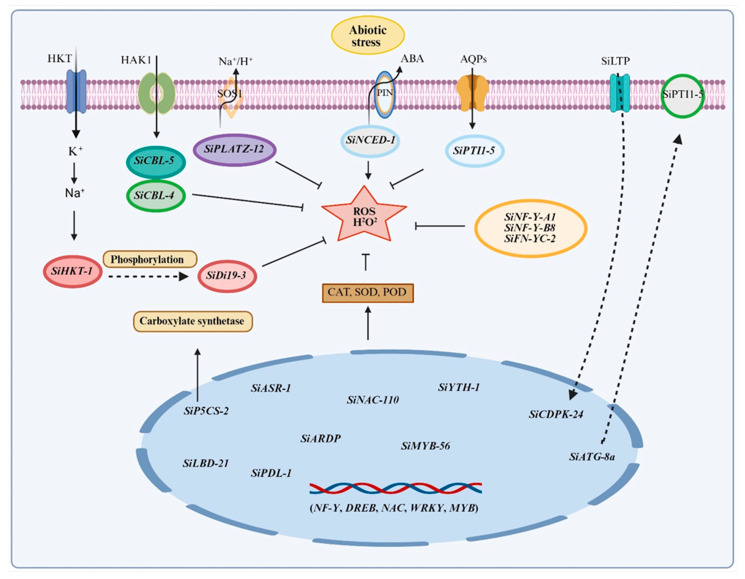
Abiotic stress response and signaling pathways in foxtail millet. The diagram illustrates key molecular mechanisms underlying abiotic stress tolerance. The SOS (Salt Overly Sensitive) signaling pathway (left) involves HKT, HAK1, and Na^+^/H^+^ antiporters (SOS1), which sense stress-induced ionic imbalances and maintain cellular ion homeostasis by regulating K^+^ uptake and Na^+^ efflux. Membrane-localized receptors including ABA receptors (PYR/PYL/RCAR family, shown as PIN), aquaporins (AQPs), and lipid transfer protein (SiLTP) with SiPTI1-5 detect stress signals. ABA signaling activates *SiNCED-1* (9-cis-epoxycarotenoid dioxygenase) and SiPTI1-5. Central to the response is ROS (H_2_O_2_) homeostasis, which is negatively regulated by *SiCBL-5*, *SiCBL-4*, and *SiPLATZ-12*, positively regulated by *SiNCED-1*, and modulated by transcription factors SiNF-Y-A1, SiNF-Y-B8, and SiFN-YC-2. Phosphorylation of *SiDi19-3* by SiHKT-1 contributes to stress signaling. Antioxidant enzymes (CAT, SOD, POD) scavenge excess ROS and are upregulated by carboxylate synthase activity. Nuclear transcription factors (*NF-Y*, *DREB*, *NAC*, *WRKY*, *MYB*) regulate the expression of downstream stress-responsive genes including *SiASR-1*, SiP5CS-2, *SiLBD-21*, *SiPDL-1*, *SiARDP*, *SiNAC-110*, *SiMYB-56*, *SiYTH-1*, *SiCDPK-24*, and *SiATG-8a*, which encode proteins involved in osmotic adjustment, ROS scavenging, and stress adaptation. Solid arrows indicate activation, dashed arrows indicate indirect regulation or translocation, and blunt-ended lines represent inhibition.

This review systematically summarizes millet responses to abiotic stresses, focusing on morphological and physiological adaptations, biochemical mechanisms and the molecular regulatory networks that modulate stress resistance. By combining the analysis of key transcription factor families (NF-Y, DREB, WRKY, NAC and MYB) with multi-omics insights, this information bridges the gap between fundamental stress physiology and applied breeding strategies. The review elaborates on complex regulatory networks, including stress signal cascades, transcriptional regulatory mechanisms, and antioxidant defense systems that regulate plant responses to drought, salinity, high and low temperatures, waterlogging and nutrient deficiencies (Figure 1 and Figure 2; Table 1, Table 2 and Table 3). This systematic review provides a foundation for developing climate-resilient crop varieties that maintain yield under variable environments. By promoting millet cultivation on marginal lands and transferring stress-resistance mechanisms to major food crops, these strategies directly address the challenges of accelerated climate change, ultimately enhancing global food security and sustainable agricultural productivity.

## 2. Foxtail Millet: A Model Crop for Stress Biology

Due to its outstanding stress resistance, well-developed genomic resources, and evolutionary relationship with major cereal crops (including rice, maize, sorghum, and wheat), foxtail millet has become a model system for studying stress biology in cereal crops [36,37,38]. This species has a compact diploid genome (2n = 18) of approximately 490 Mbp, and is well-suited for genomic analyses due to its relatively small genome size and highly efficient transformation system. High-quality reference genome assemblies are publicly available, including the milestone assembly completed by Zhang et al. [16], which covers approximately 95% of the genome and includes 38,801 annotated protein-coding genes distributed across nine chromosomes. More recently, He et al. [39] established a comprehensive pan-genome of Setaria by assembling 110 representative genomes from a worldwide collection, encompassing 35 wild, 40 landrace, and 35 modern cultivated accessions. This pan-genome comprises 73,528 gene families and 202,884 nonredundant structural variants, and was further used to construct a graph-based reference genome, providing an invaluable resource for large-scale genetic studies, marker-assisted breeding, genomic selection, and genome editing to accelerate foxtail millet improvement [39]. Multiple genome databases, including Phytozome, the Foxtail Millet Genome Database (FmGDB), and Gramene, provide annotated sequences, gene models, functional annotations, and expression data [40]. Extensive transcriptome datasets spanning diverse stress conditions and pan-genome resources enable comprehensive gene discovery and functional characterization [41,42]. Foxtail millet exhibits outstanding drought and salt tolerance, with high water use efficiency (only 257 g of water are needed to produce 1 g of biomass) [43]. In foxtail millet, stress resistance is achieved through the coordinated action of expanded transcription factor families (ARFs, WRKYs, and AP2/ERFs), an enhanced antioxidant system (SOD, APX, CAT), and osmoprotectant biosynthesis pathways [36,44,45]. Other molecular contributors include late embryogenesis abundant (LEA) proteins and stress-responsive non-coding RNAs, which together support the crop’s broad adaptability to drought, salt stress, and extreme temperatures [35].

### Role in Stress Biology Research

Millet serves as a bridge between model species and cereal crops. Transgenic studies have demonstrated that overexpression of the foxtail millet drought-induced transcription factor *SiMYB56* in rice significantly enhances drought tolerance by regulating lignin biosynthesis and ABA signaling pathways, confirming the conservation of stress-resistance mechanisms across gramineous crops [46]. This species makes genome-wide association studies (GWAS) possible, linking genomic variations to stress resistance, supporting the systems biology integration of multi-omics data, and serving as a C_4_ photosynthesis model under stress conditions [26,47,48,49]. Its transformability and the ease of genome editing make it possible to functionally validate candidate genes before extending them to major cereals with longer generation times [50]. The combination of this comprehensive genomic resource, well-characterized stress adaptability, and validated transformation potential solidifies millet status as an important model for functional genomics and crop improvement in response to climate stress.

## 3. Morpho-Physiological Adaptations to Abiotic Stress in Foxtail Millet

### 3.1. Drought Stress Adaptations

Foxtail millet exhibits significant physiological and molecular plasticity in response to drought stress, which is a key component of its adaptation strategy (Figure 1; Table 1). Under conditions of limited water, this species displays various coordinated adaptive responses at both the organ and cellular levels. The primary drought response is to minimize water loss caused by transpiration by reducing leaf surface area, which is achieved through changes in stomatal density and distribution, leaf curling and adjustments in leaf angles [51,52]. These changes effectively reduce the transpiring surface area while maintaining photosynthetic capacity.

**Table 1:** Molecular components associated with abiotic stress tolerance in foxtail millet.

Functions	Regulatory Way in Abiotic Stress	References
Acetyl-CoA carboxylase	Resistance to sethoxydim herbicide	[44]
Nitrate transporters *SiNRT* Ammonium transporters *SiAMT*	Nitrate and ammonium uptake and transport	[51]
SET domain genes	Abiotic stress tolerance	[53]
PHT1 gene family	Phosphate transporters	[54]
Argonaute protein 1 encoding gene	Regulation of stress responses	[55]
Abscisic acid stress ripening gene ASR	Drought tolerance	[56]
Autophagy related gene ATG	Tolerance to nitrogen starvation and drought stress	[57]
Late embryogenesis abundant protein (LEA)	Salt and drought tolerance	[58]
ABA responsive DRE-binding protein ARDP	Salt tolerance	[59]
SiWD40	Associated with dehydration stress-responsive pathway	[60]
Dehydration-responsive element binding protein 2 DREB2	Dehydration tolerance	[61]
NAC transcription factor	Salt tolerance	[62]
Si69	Aluminum tolerance	[63]
Aldose reductase	Associated with salinity stress-responsive pathway	[64]
Glutamine synthetase Pyrroline-5 carboxylate reductase 12-oxophytodienoic acid reductase OPR1	Drought and salt tolerance	[64]
Photosystem-II D1protein	Atrazine resistance	[65]
Phospholipid hydroperoxide glutathione peroxidase PHGPX	Associated with salinity tolerance	[66]
Nuclear factor-Y (*SiNF-YA1*, *SiNFYB8*)	Salt and drought tolerance	[67]

Meanwhile, the roots undergo key structural changes, forming a deeper and wider root network, while lateral root branching and root hair density increase. Under drought conditions, the root-to-shoot biomass ratio increases, indicating that resources are preferentially allocated to structures for water uptake rather than photosynthetic tissues [68,69]. Millet shows better water use efficiency under drought conditions compared to other cereal crops such as wheat, corn, and sorghum. Water use efficiency is optimized through coordinated physiological regulation to balance biomass production with water consumption, including reducing the transpiration rate, enhancing stomatal regulation, and increasing photosynthetic efficiency per unit of water consumed [70,71].

This outstanding performance is attributed to a unique osmotic regulation mechanism. Under drought stress, plants accumulate compatible solutes, including proline, betaine, soluble sugars, and polyols. The accumulation of these osmolytes can reduce the osmotic potential of cells, promote water influx, and maintain turgor pressure, thereby ensuring the pressure necessary for cell expansion and metabolic activities. The accumulation of proline is particularly significant, helping cells retain water and regulate osmotic pressure to sustain growth under limited water conditions [70,71,72]. These osmoprotectants also protect membrane integrity and reduce oxidative damage by scavenging ROS [73,74]. At the cellular level, plants under drought stress form thickened cell walls and enhance cuticular wax deposition, providing additional protection against water loss. Leaf tissues show an increase in trichome density, further reducing transpiration [71].

### 3.2. Salinity Stress Adaptations

Millet exhibits significant salt tolerance through morphological and physiological adaptations, allowing it to continue growing even when soil salinity increases. Under saline–alkaline conditions, its root growth remains relatively stable compared to salt-sensitive cereals, although overall biomass may decrease [75,76]. The root system shows selective changes, including lateral root development and root diameter variations, to achieve a balance between water absorption and sodium exclusion [77,78,79]. The regulation of stomatal aperture balances water conservation and photosynthetic carbon assimilation, and even under high salinity that reduces soil water potential, it helps maintain favorable water status [51,80]. Similar to responses to drought, millet regulates osmotic pressure under salt stress by accumulating soluble compatible solutes and this species shows stronger ability compared to wheat, maize and sorghum [70,77,81]. Under salt stress, the dual function of osmotic regulating substances is particularly important, maintaining cellular water balance while protecting cell structures from ion toxicity. This species demonstrates a stronger ability to sequester toxic ions in vacuoles [73,74], thereby maintaining ion ratios in the cytoplasm, which is necessary for metabolic functions.

### 3.3. Heat Stress Adaptations

Foxtail millet exhibits significant heat tolerance through coordinated morphological and physiological regulation, thereby maintaining growth and reproductive success under high temperatures [82,83]. Under heat stress, foxtail millet shows adaptive leaf responses, including increasing leaf angles and adjusting leaf orientation to reduce direct solar radiation absorption, lowering excessive heat load while maintaining photosynthetic activity [84,85,86,87]. The morphology of plant leaves may also change, with reduced leaf expansion to decrease the surface area that absorbs heat.

The composition of the cell membrane adjusts to maintain fluidity and prevent damage caused by high temperatures, with changes in lipid saturation being particularly important [66,72,73,75]. Enhanced protein stabilization mechanisms can prevent protein denaturation and aggregation, maintaining cellular metabolism under heat stress. The activity of antioxidant enzymes increases to alleviate heat-induced oxidative damage, while the accumulation of compatible solutes further stabilizes proteins and cell membranes. These comprehensive adaptive capabilities allow millet to maintain high yields even under temperatures that significantly negatively affect other cereal crops [81,85,88,89,90].

### 3.4. Water Relations and Osmotic Regulation

Water use efficiency (WUE) and osmotic adjustment are the foundations of a plant’s ability to cope with abiotic stress. Compared with cereals such as wheat, maize, and sorghum, millet exhibits superior water use efficiency and osmotic adjustment under drought and salt stress, which is related to better maintenance of cell water potential and reduced damage caused by stress [70,71,77,91,92]. This physiological advantage is attributed to unique morphological adaptations, including dense root systems to enhance water absorption, reduced leaf area to decrease transpiration, and thickened cell walls along with reinforced epidermal development to provide osmotic protection [71].

This species maintains cellular water balance by accumulating compatible solutes (proline, betaine, soluble sugars), which lower the osmotic potential of cells, thereby promoting water retention and maintaining turgor pressure [70,71,72]. Under salt stress, physiological mechanisms maintain favorable cytoplasmic ion ratios by selectively absorbing potassium, sequestering sodium in vacuoles, and restricting sodium transport in the xylem, thereby maintaining the optimal K^+^/Na^+^ ratio crucial for enzyme function [80,81].

## 4. Root Architectural Modifications and Morphological Adaptations Under Nutrient Deficiency, Drought, Salinity, and Heat Stress

Millet exhibits significant morphological plasticity in response to abiotic stresses, with the root system as the primary adaptation site [68]. These changes represent shared adaptive mechanisms across drought, salinity, heat, and nutrient deficiency, optimizing resource acquisition under different limitations.

### 4.1. Nitrogen Deficiency Adaptations

Under nitrogen-limited conditions, millet improves both nitrogen uptake and use efficiency [93,94]. Unlike nitrogen-fixing soybean, millet expands its primary and lateral root network to increase surface area for soil nitrogen acquisition [95]. Notably, under nitrogen deficiency, foxtail millet’s specific root length can reach over 46,000 cm per gram of root dry weight—ten times that of maize seedlings under the same conditions [68,96,97]. This root proliferation is accompanied by increased average root diameter and expanded vascular tissues, promoting efficient transport of nitrogen compounds [95,98]. Millet also maintains higher carbon-to-nitrogen ratios and an increased root-to-shoot dry weight ratio, reflecting an optimized carbon allocation strategy that invests more resources in nutrient-acquiring structures [68,97].

### 4.2. Phosphorus Deficiency Adaptations

Phosphorus (P) is nearly immobile in soil, requiring physical root capture. Under P deficiency, millet prioritizes lateral root elongation and increases root hair density and length, maximizing soil exploration per unit biomass [99,100]. This topsoil foraging strategy exploits bioavailable P concentrated in upper soil horizons, with reduced primary root growth redirecting carbon to fine lateral roots [101]. Cortical aerenchyma formation reduces root maintenance costs by replacing active cortical cells with air spaces, increasing root length deployable per unit carbon—an adaptation also seen under drought [100]. Increased exudation of organic acids (malate, citrate) from root tips enhances P solubilization [102]. These adjustments substantially improve P acquisition in P-impoverished marginal soils.

### 4.3. Zinc and Micronutrient Deficiency Adaptations

Zinc (Zn) deficiency induces reduced root elongation with compensatory root hair proliferation [103]. Foxtail millet adapts via enhanced exudation of phytosiderophores and organic chelators that mobilize Zn, a mechanism paralleling P-deficiency responses but targeting distinct nutrient pools [103]. Uptake occurs through ZIP-family transporters. Boron (B) deficiency impairs cell wall cross-linking, causing stunted, thickened roots [104]. Iron (Fe) deficiency induces Strategy II responses in graminaceous species, releasing mugineic acid-family phytosiderophores that chelate Fe^3+^ prior to uptake via Yellow Stripe-Like transporters [105]. These diverse responses reflect millet’s broad root morphological flexibility.

### 4.4. Root Adaptations Under Drought Stress

Under drying soil conditions, millet shifts toward deeper root development, with primary and nodal roots extending into moister layers, hydrotropic foraging [106]. This is accompanied by reduced lateral branching in surface soils, reallocating carbohydrates to deep roots [107]. Drought-stressed roots develop enlarged cortical aerenchyma, reducing metabolic costs and increasing root length per unit carbon invested, primarily to sustain water extraction [100,108]. Reductions in cortical cell number and size further decrease respiratory costs [108,109]. Enhanced root suberization (endodermis and exodermis) limits apoplastic water loss and maintains hydraulic pressure [110]. Aquaporin expression (PIPs, TIPs) regulates root hydraulic conductivity [111]. Compatible solutes (proline, glycine betaine, sugars) accumulate in roots, maintaining osmotic gradients for water absorption [112]. The higher root-to-shoot ratio under drought, compared to major cereals, reflects an inherent capacity to prioritize root biomass investment under water limitation [113]. Collectively, these root-level morphological, anatomical, and physiological adjustments position foxtail millet as a model species for studying adaptive root plasticity under combined nutrient and water stress.

### 4.5. Root Adaptations Under Salinity Stress

Salinity imposes both osmotic stress (similar to drought) and ionic toxicity. Under saline conditions, foxtail millet exhibits reduced primary root elongation, while lateral root density and root hairs are maintained or modestly enhanced to sustain water and nutrient uptake [75,77]. This contrasts with drought, where primary root elongation is promoted. Root anatomical adaptations include enhanced suberin deposition in endodermal and exodermal layers, restricting apoplastic Na^+^ entry into the stele and limiting xylem loading [110]. The Casparian strip is reinforced, providing a tighter apoplastic barrier. At the cellular level, Na^+^ is sequestered into vacuoles via tonoplast-localized NHX antiporters (SiNHX1, SiNHX2), energized by vacuolar H^+^-ATPase [114]. High-affinity potassium transporters (SiHKT1) limit Na^+^ influx while maintaining K^+^ uptake, preserving the cytosolic K^+^/Na^+^ ratio [115]. Cortical aerenchyma is also induced under severe salinity, reducing metabolic costs and freeing resources for ion homeostasis [100].

### 4.6. Root Adaptations Under Heat Stress

Heat stress directly impairs root meristematic activity and membrane integrity at elevated soil temperatures. Under moderate heat stress, foxtail millet roots show reduced primary root elongation and modified gravitropism due to impaired auxin polar transport [82,88]. Root tip meristematic cells are vulnerable to heat-induced protein denaturation. Millet roots upregulate heat shock proteins (SiHSP17/SiHSP70) as molecular chaperones stabilizing root tip proteins [33,91]. Membrane lipid composition adjusts with increased fatty acid unsaturation to maintain fluidity. Compatible solutes (proline, glycine betaine) accumulate in root tissues, stabilizing membranes and enzymatic function at high temperatures [12,70]. Unlike drought, heat stress may constrain root depth due to elevated deep-soil temperatures, shifting root distribution toward shallower, cooler layers as a thermotropic avoidance response. Heat-induced root senescence in older sections is partially counteracted by new root primordia from the shoot base [82,91].

Collectively, foxtail millet’s superior performance under multiple abiotic stresses stems from conserved adaptive strategies deployed in stress-specific configurations. Modified root architecture improves resource acquisition under nutrient deficiency and drought; reduced leaf area and stomatal regulation minimize water loss under drought and salinity while enabling cooling under heat; osmotic adjustment through compatible solutes functions across drought, salinity, and heat; and cellular protection via membrane stabilization and antioxidant capacity is recruited across all stress types. These integrated cross-stress adaptive traits position foxtail millet as a resilient cereal capable of maintaining productivity under diverse and often concurrent environmental constraints.

## 5. Biochemical Adaptations to Abiotic Stress

### 5.1. Osmotic Adjustment 

Osmotic adjustment maintains turgor and metabolism under abiotic stress by accumulating compatible solutes such as proline, betaine, and sugars. These solutes lower the osmotic potential to facilitate water influx [73,112,116]. These osmoprotectants provide dual protection by maintaining membrane integrity and scavenging ROS [73,117]. In millet, the accumulation of these substances, especially proline, can enhance water retention and redox balance, thereby maintaining growth under drought conditions [70,71,72]. The concentration of osmoprotectants in stress-tolerant genotypes is consistently significantly higher than in sensitive varieties.

Compared with major cereal crops such as wheat, corn and rice, millet shows a significant advantage in osmotic regulation. Under the same drought conditions, millet accumulates proline and other osmotic regulators much faster than these crops, allowing it to reduce osmotic potential more quickly and better retain water [70,71,72]. This superior ability is attributed to millets stronger and faster upregulation of key biosynthetic genes (such as *SiP5CS-2* for proline synthesis and *SiBADH* for betaine synthesis) compared to drought-sensitive cereals. In addition, drought-tolerant millet genotypes can maintain higher concentrations of osmolytes under prolonged stress, thereby sustaining growth and photosynthesis, whereas sensitive varieties are damaged by dehydration [70,71,72]. This enhanced osmotic regulation capability is the key factor behind millet’s outstanding drought resistance and its ability to produce grain under water-scarce conditions where other cereals cannot grow.

### 5.2. Nitrogen Redistribution and Internal Transport

After nitrate absorption, efficient redistribution from roots to stems and from mature leaves to developing leaves is crucial for the nitrogen economy of plants [118]. In Arabidopsis, NRT1.11/NRT1.12 regulate nitrate loading from the xylem to the phloem and redistribution within leaf tissues, which occurs under normal nitrogen supply conditions [119]. In millet seedlings experiencing severe nitrogen limitation (treated with 0.02 mM ammonium nitrate for 7 days), significant adaptability is demonstrated by upregulating the expression of *SiNRT1-11*/*SiNRT1-12*, thereby enabling effective redistribution of nitrate within stem and leaf tissues. Ammonium ions supplement nitrate absorption through ammonium transporters (AMTs) [120]. Enhanced expression of *SiAMT-1.1* accelerates nitrogen uptake in nitrogen-limited millet [68], contributing to an overall improvement in nitrogen use efficiency.

### 5.3. Antioxidant Response-Based Abiotic Stress Tolerance

Abiotic stress can lead to excessive accumulation of ROS, such as H_2_O_2_ and O_2_^−^, resulting in cellular oxidative damage [85,121]. Plants mitigate this damage through a dual antioxidant system that includes enzymatic components (SOD, CAT, APX) and non-enzymatic antioxidants (GSH, ascorbate, flavonoids) (Pan). In millet crops, tolerance is closely related to the efficiency of this system. Under stress, tolerant genotypes consistently exhibit increased SOD, CAT, and APX activities, along with decreased lipid peroxidation (MDA) levels, compared to sensitive varieties [43,51].

This relationship has been functionally verified through transgenic research. Overexpressing maize genes such as *SiLEA14*, *SiNRX1*, *SiNF-YA1* and *SiASR4* in heterologous systems can enhance stress resistance by increasing the activity of reactive oxygen species-scavenging enzymes and reducing oxidative markers [51,57,122,123]. For example, the SOD gene family in millet includes eight members, among which specific isoforms such as *SiCSDs* and *SiMSD* can be expressed up to 20 times higher under stress conditions [124]. Complementary osmotic regulation and non-enzymatic pathways also play key roles; the proline biosynthesis enzyme P5CS (especially the *SiP5CS-2* isoform) is strongly induced under osmotic stress [72], while enhanced cysteine biosynthesis via OASTL supports glutathione production for ROS detoxification [54,125,126,127]. These interconnected pathways together form an integrated regulatory network that supports the stress resistance of maize (Figure 2; Table 2).

**Table 2:** Genes and proteins associated with the reduction of ROS levels in the abiotic stress response of foxtail millet.

Gene Name	Name of Protein/TF	Regulatory Way in Abiotic Stress	Reference
*SiP5CS-2*	Δ1-pyrroline-5 carboxylate synthetase	Promotes proline accumulation.	[72]
*SiATG-8a*	Autophagy-related gene	Increases chlorophyll and proline contents and reduces MDA content	[94]
*SiASR4*	Abscisic acid stress repining	Enhances SOD and CAT activities and reduces ROS accumulation	[122]
*SimiR396d*	MiRNA	Regulates root growth.	[125]
*SiYTH-1*	YTH	Reduces H_2_O_2_ accumulation.	[126]
*SiMYB19*	MYB	Reduces ion leakage, chlorosis, and growth inhibition.	[127]
*SiLBD21*	Lateral organ boundaries domain LBD	Regulates plant growth and development under drought stress	[128]
*SiZAT12*	ZAT	Regulates proline biosynthesis, ion homeostasis	[129]
*SiNCED-1*	9-cis-epoxycarotenoid dioxygenase NCED	Modulates ABA biosynthesis and enhances and SOD and CAT activities	[130]
*SiCDPK-24*	Calcium dependent protein kinases CDPKs	Enhances drought tolerance	[131]
*SiPYL3*	PYL (Pyrabactin resistance 1-like)	Plays a significant regulatory role in abiotic stress responses	[132]
*SiNAC-110*	NAC	Regulates proline biosynthesis, ion homeostasis	[133]

## 6. Transcription Factor Families Mediating Stress Tolerance

**Figure 3:**
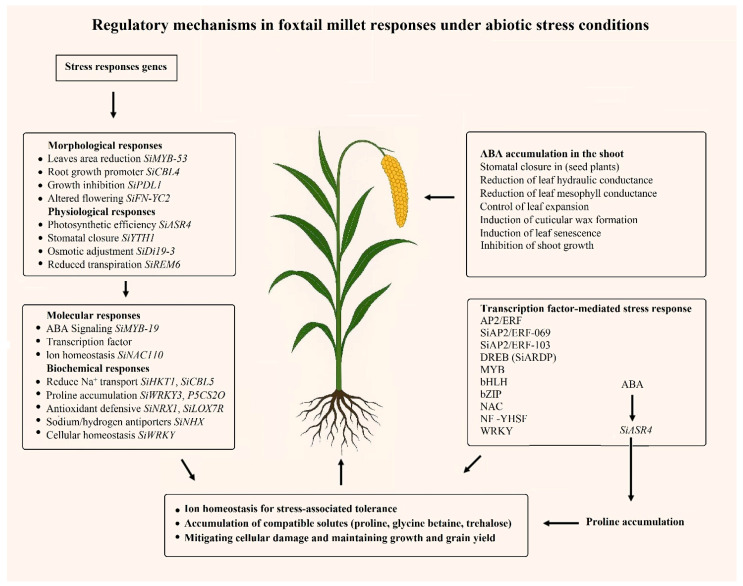
Abiotic stress response network in foxtail millet. Stress tolerance is achieved through the coordinated expression of genes regulating ion homeostasis, osmotic adjustment, oxidative defense, and cellular protection. Key regulatory genes are indicated in parentheses.

With the help of recent innovations in sequencing technology, transcriptomic research on foxtail millet has deepened the understanding of known and novel abiotic stress tolerance mechanisms, particularly regarding key genes and pathways (Figure 3; Table 3). During environmental stress conditions, plants activate transcription factors to regulate gene expression through interactions with cis-regulatory elements located in promoter and enhancer regions [41,132]. For example, recent studies have shown that transcription factor-mediated gene regulation constitutes a fundamental mechanism by which plants respond to environmental stresses, providing key insights into adaptive responses at the molecular level. Several transcription factor families play crucial roles in millet stress resistance mechanisms, including *DREB* (dehydration responsive element binding protein), *MYB* (myeloblastosis), *NAC*, *NF-Y* (nuclear factor Y), *HSF* (heat shock factor), *WRKY*, and *AP2/ERF* (APETALA2/Ethylene Responsive Factor) families [83,85,86,132,133]. Collectively, these transcription factors operate through interconnected regulatory networks, establishing a robust molecular framework that facilitates rapid and coordinated stress responses, thereby enhancing stress tolerance (Figure 1 and Figure 3; Table 3).

The AP2/ERF family exhibits significant diversity in stress response mechanisms. Genome-wide in silico analysis has identified *SiAP2/ERF-069*, *SiAP2/ERF-103*, and *SiAP2/ERF-120* as pivotal regulators that enhance abiotic stress tolerance through ABA-dependent signaling pathways [44]. The NAC and DREB families contribute through both ABA-dependent and ABA-independent mechanisms. *SiNAC-110* shows notable stress-induced expression patterns and, when overexpressed in transgenic Arabidopsis, enhances stress tolerance by modulating genes involved in proline biosynthesis, ion homeostasis, and osmotic regulation through ABA-independent mechanisms [61,133]. Similarly, the *DREB* family interacts with DRE/CRT cis-elements in stress-responsive gene promoters. Overexpressing the *SiARDP* gene in Arabidopsis is an example of this mechanism, as it significantly enhances abiotic stress tolerance during seed germination and seedling growth [115]. In addition, SiARDP interacts with SiASR-4 at the protein level, leading to increased expression of *SiASR4* and activating stress response genes and ROS scavenging pathways through an ABA-dependent signaling cascade [122].

**Table 3:** Genes of transcription factor family members associated with abiotic stress tolerance in foxtail millet.

Gene Name	Name of Protein/TF	Regulatory Way in Abiotic Stress	Reference
*SiSAUR*	LOXs	Promote antioxidant enzyme activities.	[30]
*SiWRKY*	WRKY	Reduces oxidative stress and proline contents.	[36]
*SiPLATZ12*	Platz	Negatively regulates drought tolerance by lowering Na+/H+ antiporter activities of SOS1 and NHXs	[48]
*SiNF-Y-A1*	Nuclear Factor Y	Enhances abiotic tolerance and antioxidant system and reduces ROS	[51]
*SiLTP*	Nonspecific lipid transfer protein	Involved in the ABA-dependent transduction signal pathways. Promotes proline and soluble sugar content.	[72]
*SiP5CS-2*	Δ1-pyrroline-5-carboxylate synthetase	ROS scavenging.	[80]
*SiARDP*	DREB	Promotes proline accumulation	[115]
*SiASR-4*	Abscisic acid stress and ripening induction	Reduce ROS accumulation.	[122]
*SiMYB19*	MYB	Promotes primary root growth.	[127]
*SiLBD21*	LBD	Stress response	[128]
*SiNAC-110*	NAC	Regulates proline biosynthesis, ion homeostasis, and ABA signaling pathway-independent osmotic balance	[133]
*SiNF-YC2*	Nuclear Factor Y	Enhances drought tolerance and reduces ROS accumulation	[134]
*SiDi19-3*	Cys2/His2-type	Upregulates NHX, SOS, and CBL genes under drought stress.	[135]
*SiARDP*	REM	Reduces electrolyte leakage and increases proline accumulation.	[136]
*SiPLT-L1*	PTI1	Regulates dynamic ROS balance.	[137]
*SiLTP*	PTI1	Plays important roles in improving stress tolerance	[138]
*SiCBL-4*	Calcineurin B-like protein	Enhances root growth.	[139]
*SiCIPK-24*	CIPK	Promotes root elongation.	[139]
*SiCEP-3*	C-terminal-encoding peptides	Promote ABA import.	[140]
*SibZIP-67*	bZIP	Reduces malondialdehyde, enhances antioxidant enzyme activities.	[141]
*SiWRKY-3*	WRKY	Reduces oxidative stress and proline contents.	[142]

The NF-Y family constitutes an important regulatory system for stress resistance in maize [51]. Recent studies on the overexpression of *SiNF-Y-A1*, *SiNF-Y-B8* and *SiNF-Y-C2* genes in tobacco have shown that it significantly enhances drought and salt tolerance [51]. These transgenic lines exhibit excellent physiological performance under stress conditions, including stable relative water content, maintained chlorophyll concentration, and increased activities of key antioxidant enzymes (SOD, POD, and CAT), while MDA levels decrease, indicating reduced cellular membrane damage (Table 3). Overall, the transcription factor network in maize enhances the ability to respond to oxidative stress and provides protection against lipid peroxidation under adverse conditions. This mitigation of cellular damage helps maintain growth and grain yield under environmental stress.

### Molecular Mechanisms of Stress Tolerance

Millet exhibits strong tolerance to abiotic stress through coordinated genetic and physiological adaptations. *SiPIP*, *SiTIP*, and *SiNIP* regulate water transport, while ion transport proteins (e.g., *SiHKT1*) and regulatory kinases (e.g., *SiCIPK24*) maintain cellular ion homeostasis under drought and salt stress [115,127,133,142]. Stress-responsive transcription factors, including *SiDREB2*, *SiNAC*, *SiARDP*, *SiWRKY3*, and *SiMYB19*, coordinate the expression of downstream effector genes [32,36,115,127,133]. These target genes encode protective proteins, such as late embryogenesis abundant proteins (*SiLEA*), heat shock proteins (*SiHSP*), and enzymes that synthesize compatible solutes (*SiHSP70*), which collectively promote osmotic regulation, protein stability, and oxidative stress mitigation [57,72,143]. Taken together, these findings indicate enhanced osmotic regulation capacity and stress tolerance in foxtail millet, establishing a direct link between gene regulation and stress-resistant phenotypes (Table 1). The compact genome structure of foxtail millet further facilitates functional genomics research, accelerating the identification of stress-responsive genes and advancing our understanding of abiotic stress tolerance in cereal crops.

## 7. Multi-Omics: Molecular Detection Tools for Abiotic Tolerance in Foxtail Millet

Multi-omics approaches, including genomics, transcriptomics, proteomics, metabolomics, ionomics, epigenomics, and phenomics, as well as QTL analysis, have significantly advanced the identification of candidate genes and molecular markers associated with plant abiotic stress tolerance [30,32,48,49,144,145,146]. For example, integrative approaches elucidate the complex physiological mechanisms of cellular responses to environmental stress, providing comprehensive insights into stress adaptation strategies in millet (Figure 1, Figure 2 and Figure 3; Table 2 and Table 3).

### 7.1. Transcriptomic and Genomic Applications

RNA sequencing analysis has identified many differentially expressed genes that regulate key agronomic traits. For example, Zhu et al. [28], identified key genes controlling plant height, pinpointing the gibberellin biosynthesis gene *Seita.5G404900*, where a frameshift mutation co-segregates with the dwarf phenotype [28]. The developed molecular markers provide valuable tools for marker-assisted breeding programs aimed at improving lodging resistance. Complementing studies on plant height, Wang et al. [26], investigated the transcriptional dynamics of grain development across four developmental stages, offering valuable genetic resources and candidate genes for enhancing grain quality and yield.

Additionally, Yang et al. [32], identified 166 *SiDREB* transcription factors, highlighting their role in abiotic stress responses, particularly saline–alkali tolerance, with *SiDREB153* and *SiDREB80* serving as crucial functional candidates for crop resilience improvement.

Multiple transcription factor families contribute to stress tolerance mechanisms. *SiNAC* (*SiNAC1-2*) and *SiWRKY* (*SiWRKY-12*) transcription factors exhibit stress-inducible expression patterns and confer enhanced tolerance when overexpressed in transgenic systems [36,133,142]. Nuclear Factor-Y (*NF-Y*) genes function as critical regulatory components, showing rapid evolutionary expansion under strong purifying selection pressures [51]. Stress inducible genes *SiNF-Y-A1* and *SiNF-YB8* act as transcriptional activators, with expression positively regulated by ABA and H_2_O_2_ signaling pathways under drought and salt stress conditions. Zhang et al. [30], systematically identified 70 *SiSAUR* genes, revealing their roles in hormone signaling and stress response. 10 *SiSAUR*s are induced by abiotic stress and exogenous hormones, confirming their involvement in auxin-mediated physiological processes and supporting previous proteomic findings on *SiSAUR*s role in plant height regulation. Xiao et al. [48] identified *SiPLATZ-12* as a key negative regulator of salt tolerance, directly repressing critical ion homeostasis genes *SiNHX-2* and *SiCBL-4*. Natural variation in its promoter between cultivated and wild genotypes highlights a potential domestication site for enhancing salt tolerance through molecular breeding. Further, given that foxtail millet is susceptible to cold, future multi-omics studies should investigate cold-responsive mechanisms, including the role of stress proteins (SiDHN dehydrins, LEA, and HSPs) and transcription factors (e.g., *SiDREB*, *SiNAC*, *SiWRKY*) in enhancing freezing and chilling tolerance [32,33,51,133,142].

### 7.2. Proteomic Investigations

Mass spectrometry-based proteomic investigations have characterized stress-response proteins accumulating during abiotic stress exposure (Supplementary Table S1). For example, Guo et al. [33], demonstrated that dehydrin proteins of the *SiDHN* gene significantly enhance tolerance to freezing and drought stress in transgenic plants, establishing SiDHN as a promising candidate for genetic engineering of stress-resilient crops. Additional key proteins include late embryogenesis abundant proteins (SiLEA-1, SiLEA-3) and heat shock proteins (SiHSP17, SiHSP70). Qin et al. [35] characterized 94 *SiLEA* genes, identifying ABA responsive members *SiASR-2*, *SiASR-5*, and *SiASR-6* as potential key regulators in abiotic stress tolerance. Zhao et al. [31] revealed that auxin signaling, mediated through *SiSAUR* like, *SiGH-3*, and *SiTCH-4*, constitutes a central pathway governing internode elongation and plant height. *SiSAUR*, marked by FCM1-2 molecular markers, provides a valuable tool for marker-assisted selection in dwarfing breeding programs, complementing previous findings related to gibberellins.

### 7.3. Ion Transport and Osmotic Regulation

Genomic studies have identified key ion transport genes that contribute to salt tolerance. Under saline–alkaline conditions, plants upregulate genes for sodium/hydrogen antiporters (*SiNHX*), high-affinity potassium transporters (*SiHK*) and vacuolar H^+^-ATPases (*SiVHA*) in the roots to maintain ion homeostasis. *SiNHX* proteins transport sodium ions out of the cytoplasm, while *SiHK* regulates the distribution of potassium and sodium in roots and stems. Vacuolar H^+^-ATPases maintain cellular pH and create a proton gradient to compartmentalize sodium ions via NHX antiporters [114]. Stress-tolerant varieties can maintain high concentrations of osmoprotectants under adverse conditions, including proline, betaine, and trehalose. In tolerant varieties, the accumulation of phenolic compounds, flavonoids, and organic acids increases, helping to enhance antioxidant capacity and cellular protection mechanisms [147].

### 7.4. QTL Mapping and Candidate Gene Identification

Advances in rice genomics have accelerated the identification of QTLs associated with traits related to stress resistance and yield, including panicle traits such as qGL3 (grain length), qPBN1 (primary branch number), and qSPF (spikelet fertility rate) [148]. Candidate genes include *SiDL* and *SiYAB-1* (a YABBY transcription factor), as well as homologous genes of *DEP-1*/*TB1*, which regulate branching and inflorescence architecture [149]. QTL mapping has identified genomic regions associated with drought and salt tolerance, including major QTL affecting root architecture, water use efficiency, and osmotic regulation. These QTL co-locate with genes encoding aquaporins (*SiPIP1-2*), enzymes for osmolyte synthesis, and stress-responsive transcription factors, providing valuable targets for marker-assisted selection [32,150].

**Figure 4:**
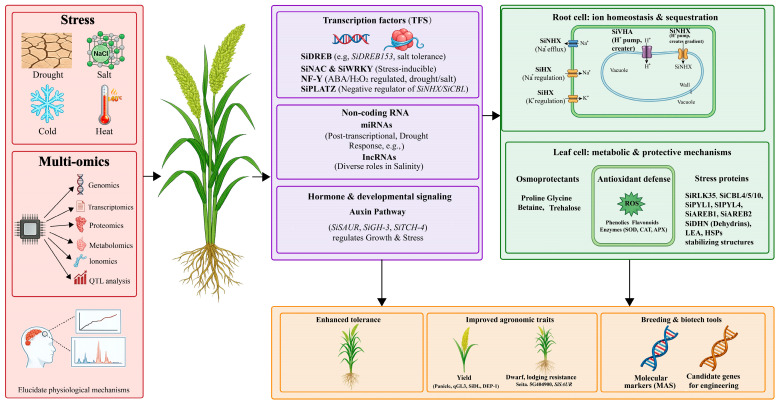
Multi-omics integration for foxtail millet improvement under abiotic stress conditions. The central diagram depicts foxtail millet exposed to major abiotic stresses (drought, salt, cold, and heat) that significantly impact plant growth and yield. Surrounding sectors represent different omics approaches genomics, transcriptomics, proteomics, metabolomics, ionomics, and QTL analysis, with their associated stress-responsive genes and molecular markers. Cross-talk arrows demonstrate the interconnected nature of these omics layers in elucidating stress tolerance mechanisms. Root cells show ion homeostasis and sequestration pathways (SiNHX, SiHX, SiVHA), while leaf cells highlight metabolic and protective mechanisms including osmoprotectants, antioxidant defense, and stress proteins. Integration of multiple omics datasets from various plant tissues under single or combinatorial stress conditions generates comprehensive molecular profiles essential for developing climate-resilient crops through enhanced tolerance, improved agronomic traits, and breeding/biotech tools.

Multi-omics integration provides a comprehensive understanding of the genetic control mechanisms underlying millets superior stress resistance (Figure 4). These findings offer valuable genetic resources for crop improvement and the development of climate-resilient varieties through conventional breeding and biotechnological methods, thereby facilitating the translation of molecular research into practical agricultural applications.

### 7.5. Non-Coding RNAs

Non-coding RNAs play a central role in gene regulation in foxtail millet. Das et al. [151] identified 136 high-confidence miRNAs and 2417 target genes across seven tissues during the jointing and grain-filling stages. They also constructed an miRNA-centered integrated regulatory network. This network, which contains feedforward loop motifs at its core, integrates upstream transcription factors and downstream target genes, providing a foundation for understanding miRNA functions in foxtail millet [151].

Long non-coding RNAs (lncRNAs) have also been characterized in foxtail millet. Zhang et al. [30] identified 12,378 novel lncRNAs in young spikelets from varieties with different yield levels. Among these, 70 were differentially expressed between high-yield and conventional varieties, with 67 predicted to regulate target genes in cis or in trans, and 18 functioning as miRNA target mimics [152]. While these studies focused on yield regulation, future research should explore stress-responsive non-coding RNAs and their interactions with transcription factors such as DREB and NAC to fully understand millet’s abiotic stress tolerance.

### 7.6. Ionomics Applications in Tolerance Enhancement Under Abiotic Stress

Ionomics, which is the comprehensive analysis of the elemental composition in biological systems, has become a powerful tool for improving crop cultivation under abiotic stress [153]. By systematically analyzing the elemental composition of plants, this omics approach provides mechanistic insights into nutrient homeostasis disorders and metal toxicity responses [154]. Anas et al. [155], recently employed integrated physiological and ionomic approaches to examine nickel (Ni) stress in two wheat varieties (SKD-1 and Borlaug-16) under controlled conditions (100 mg L^−1^ Ni for 21 days). Ion analysis revealed different Ni migration patterns: SKD-1 showed enhanced Ni translocation to the aerial parts due to reduced root retention, while Borlaug-16 exhibited better Ni sequestration in the roots. Elemental distribution analysis further showed that Borlaug-16 accumulated higher concentrations of trace elements (Mn, Zn, Pb, Cr, Cu) in the roots, whereas SKD-1 accumulated more essential nutrients (Fe, Ca, Mg, Na, P) in the leaves. Morphologically, Borlaug-16 showed increased root cortex and vascular bundle thickness, reduced oxidative stress markers, and elevated antioxidant enzyme activities (SOD, CAT, APX). In contrast, SKD-1 suffered severe oxidative cellular damage. The reduced nickel uptake in Borlaug-16 and its strong antioxidant defense system indicate superior stress resistance, highlighting the potential of developing sustainable crop improvement strategies under metal pollution conditions through ionomics-guided approaches.

## 8. Foxtail Millet as a Genetic Resource for Panicle Resilience

Foxtail millet is a promising model system for dissecting the genetic basis of panicle resilience (the ability to maintain reproductive development and grain set under stress). This is due to its fully sequenced genome and the diverse germplasm resources, including landraces, mutants, and recombinant inbred lines, which enable robust genetic analyses. As a result, QTL mapping and GWAS have successfully identified genomic regions that control panicle architecture and fertility under both optimal and stress conditions [33,133,149]. Several key candidate genes regulating these stress-adaptive traits have recently been identified (Figure 3; Table 1, Table 2 and Table 3). For instance, NAC transcription factors, such as *SiNAC-110* discovered via GWAS, promote panicle branching and floral meristem activity under abiotic stress [133]. Similarly, *SiYAB* genes are highly expressed in reproductive tissues, and the overexpression of *SiDL* reduces seed size, implying that natural allelic variation at these loci could underpin panicle trait variation [149]. As these panicle traits co-localize with discrete QTLs, the *SiYAB* family-particularly SiDL constitutes a prime set of positional candidates for those loci. Integrating this *YABBY* expression and functional data with existing QTL maps will thus accelerate the identification of causal genes [149]. In addition, the *SiDHN* gene significantly enhances drought resistance [33]. This is consistent with genes frequently upregulated in the spike that are involved in broader protective mechanisms, such as osmolyte synthesis, reactive oxygen species (ROS) scavenging, and heat shock responses during stress, suggesting that they play a key role in protecting reproductive development (Figure 2; Table 2).

### 8.1. Breeding Strategies and Applications

Improving ear traits provides a direct way to enhance yield stability under abiotic stress conditions. Modern maize breeding programs increasingly emphasize combining phenotypic selection with marker-assisted selection and genomic approaches. Current work focuses on: (i) ear extension angle, which affects pollination efficiency under temperature fluctuations; (ii) compact and high-yield branching structures, optimizing resource allocation; and (iii) the fertility of heat-tolerant pollen, a key determinant of grain formation under heat stress. These traits offer measurable and heritable targets for field selection [156].

### 8.2. Gene Editing Applications

Emerging gene editing technologies, such as CRISPR-Cas9, have facilitated precise molecular interventions in millet breeding programs through various targeted approaches. For example, researchers used CRISPR/Cas9-mediated base editing to directly confirm the role of *SiGS1* in glyphosate tolerance, demonstrating that a single, precise amino acid residue edit (S59G-T60A) can confer herbicide resistance to foxtail millet without affecting normal agronomic traits [157]. This proof of concept demonstrates that modern gene editing can quickly introduce or fine-tune beneficial alleles without bringing the additional genetic burden associated with traditional breeding, thereby accelerating the improvement of high-quality millet varieties in cereal metabolomics and anti-inflammatory traits [22].

CRISPRa provides a method for millet breeders that may be considered non-transgenic under certain regulatory frameworks, by precisely upregulating the endogenous *SiERFV* gene without altering its coding sequence, thereby enhancing flood tolerance, allowing crops to be cultivated in flood-prone areas while maintaining a favorable agronomic trait background [158]. *SiEPF-2* illustrates how CRISPR/Cas9-mediated single peptide ligand site editing can simultaneously fine-tune stomatal density to enhance drought resistance and remodel inflorescence structures to increase yield, providing breeders with a precise, multi-gene-free method to stack these traits in elite millet lines [159]. Targeted editing of inflorescence development genes, including the APO1 orthologs and related structural regulatory genes, can achieve controllable regulation of spikelet density, branching patterns, and floral determinacy, thereby optimizing grain yield potential under stress conditions. Strategic regulation of genes involved in plant hormone biosynthesis and signal transduction, especially those controlling auxin transport and cytokinin metabolic pathways, makes it possible to finely adjust the morphological traits of the inflorescence, including size, compactness, and developmental timing. Functional validation of these genomic targets through systematic knockout and knock-in experiments will accelerate the development of climate-resilient ideotypes with enhanced panicle architecture, improved resource allocation efficiency, and optimized reproductive success under diverse environmental stress scenarios. The CRISPR/Cas9 single- and multi-gene knockout system targets the *SiFMBP*, *SiDof-4*, *SiBADH-2*, *SiGBSS-1* and *SiIPK-1* genes in foxtail millet protoplasts to screen for highly efficient targeted sgRNAs. Subsequently, homozygous mutant plants with most of the targeted genes were recovered through Agrobacterium-mediated genetic transformation of foxtail millet [23]. The mutagenesis frequency in the T0 generation was as high as 100%, and it was stably passed on to the next generation [160]. After screening these targeted edited events, no off-target mutations were detected at potential sites. Based on this system, base editing has been successfully achieved using two base editors (CBE and ABE) to target the *SiALS* and *SiACC* genes of foxtail millet [23]. By utilizing CBE to target the *SiALS* gene, a homozygous herbicide-tolerant mutant plant was created. The current system could enhance the analysis of functional genomics and genetic improvement of foxtail millet.

## 9. Integration and Future Perspectives

The abiotic stress resistance of millet originates from an integrated regulatory network encompassing morphological plasticity, biochemical adaptability, and molecular regulation (Figure 1, Figure 2 and Figure 3; Table 1, Table 2 and Table 3). This integration is evident in root structure remodeling, which not only aids nutrient acquisition but also enhances water absorption, the formation of cortical air spaces reduces metabolic costs under phosphorus deficiency and drought conditions, while lateral root proliferation increases resource-capturing capacity [100,108,161]. Osmotic adjustment through the accumulation of compatible solutes plays a role in drought, salt stress, and heat stress, with proline biosynthesis regulated by stress-responsive transcription factors (*SiARDP*, *SiNAC110*), which activate P5CS enzyme and downstream antioxidant pathways [115,122,133].

A unifying principle emerges: transcription factor networks (*DREB*, *NF-Y*, *NAC*, *WRKY*, *MYB*) are not direct stress sensors, but rather integrative hubs that convert various environmental inputs into coordinated molecular responses. Stress signals regulate gene expression through cascade signaling, thereby facilitating interactions between metabolic states, developmental programs, and stress adaptation networks. However, key questions still remain. Which specific metabolic intermediates can activate different stress response pathways in reproductive tissues? How do chromatin-modifying enzymes directly sense metabolic changes to generate tissue-specific modifications? Can precise developmental regulation optimize spike traits without compromising yield? More importantly, how do natural allelic variations contribute to differences in stress resistance between different germplasms? Addressing these questions will elucidate the fundamental design principles underlying the integration of stress, metabolism, and development in controlling spike branching, grain filling, and the maintenance of floret productivity. New technologies, including single-cell multi-omics, real-time chromatin imaging, high-throughput phenotyping platforms and systems biology modeling, will make it possible to map spatiotemporal regulatory dynamic maps and identify rational crop improvement targets. The ultimate goal is to design climate-adapted ideal crops, achieving optimized resource allocation and stress resistance, thereby enhancing the productivity of marginal lands under rapidly changing climate conditions.

## Data Availability

The authors do not have permission to share data.

## Supplementary Materials

The following supporting information can be downloaded at: https://www.mdpi.com/article/10.3390/plants15101474/s1, Table S1. Proteins involved in signaling in foxtail millet under abiotic stress conditions.
